# The Hospital Chapel

**Published:** 1908-03-14

**Authors:** L. G. Davies


### Editor's Letter-Box.

### THE HOSPITAL CHAPEL.

Sir,—I note in my copy of The Hospital for the 7th inst. a reference on p. 611 to the unfortunate *impasse* at Newcastle, but in justice to the Free Churches will you find room for the enclosed extract from the *British Weekly* of December 19, 1907, which summarises the position up to that date.

Far be it from me to introduce so-called "religious" bickering into your columns, but it should at least be known that the quarrel at Newcastle is not of the Nonconformists' seeking. Yours faithfully,

L. G. Davies, M.D.

The following is the extract referred to :—

From "British Table Talk" in the *British Weekly*, December 19, 1907.

. . . "A chapel was built in connection with the new infirmary at a cost of over £5,000 *out of the moneys publicly subscribed to the common building fund* [italics mine]. The Free Churches arranged to provide a chaplain at their own cost, and asked permission to conduct service in the new chapel. Eight days after their application this chapel was consecrated, and they were informed that permission would be given to conduct services in the students' lecture hall, but that, owing to consecration, they were excluded from the chapel. The students' lecture hall, which has proved unsuitable, was accepted under protest. The Presbytery of Newcastle thereupon felt called upon to take action on their own account against an inequitable arrangement, and pleaded for equal rights to all Churches in the use of the chapel. . . ."

